# Evaluation of ivabradine in left ventricular dyssynchrony and reverse remodeling in patients with chronic heart failure

**DOI:** 10.1002/joa3.12398

**Published:** 2020-07-05

**Authors:** Korhan Soylu, Idris Bugra Cerik, Gokhan Aksan, Gokay Nar, Murat Meric

**Affiliations:** ^1^ Department of Cardiology Faculty of Medicine Ondokuz Mayis University Samsun Turkey; ^2^ Department of Cardiology Faculty of Medicine Cumhuriyet University Sivas Turkey; ^3^ Department of Cardiology Samsun Education and Research Hospital Samsun Turkey; ^4^ Department of Cardiology Faculty of Medicine Pamukkale University Denizli Turkey

**Keywords:** dyssynchrony, heart failure, ivabradine

## Abstract

**Objectives:**

Ivabradine is a pharmacological agent used in patients with heart failure and sinus rhythm. Its only known pharmacological effect is to slow the heart rate. In this study, we investigated the impact of ivabradine on dyssynchrony parameters in heart failure patients.

**Methods:**

In this study, we assigned 55 patients taking medication for heart failure to receive ivabradine in addition (Group I). Twenty healthy volunteers comprised Group II. Echocardiographic measurements (dyssynchrony, left ventricular volumes and left ventricular ejection fraction) were taken at baseline, 1 month, and 3 months.

**Results:**

A total of 32 heart failure patients in Group I completed the study. There was significant improvement in dyssynchrony parameters after ivabradine treatment in Group I. Interventricular dyssynchrony (IVD) decreased from 42.0 ± 24.4 milliseconds at baseline to 33.6 ± 20.7 milliseconds at 1 month (*P* = .001) and to 30.7 ± 19.4 milliseconds at 3 months (*P* < .001). Septal to posterior wall motion delay decreased from 90.3 ± 21.4 milliseconds to 83.9 ± 26.9 milliseconds (*P* = .011) at 1 month and to 81.5 ± 27.3 milliseconds at 3 months (*P* = .001). Septal to lateral Ts delay (Ts‐SL) decreased from 42.7 ± 24.5 milliseconds to 35.8 ± 22.6 milliseconds at 1 month (*P* < .001) and to 34.8 ± 22.4 milliseconds at 3 months (*P* = .002). Left ventricular end‐systolic volume (LVESV) decreased from 139.4 ± 42.2 mL to 135.3 ± 39.6 mL at 1 month (*P* = .006) and to 123.3 ± 39.5 mL at 3 months (*P* < .001).

**Conclusion:**

The addition of ivabradine to heart failure treatment improves cardiac dyssynchrony parameters in chronic systolic heart failure patients with sinus rhythm.

## Graphical abstract

Ivabradine is only known pharmacological effect is to slow the heart rate. We investigated the impact of ivabradine on dyssynchrony parameters in heart failure patients. The addition of ivabradine to heart failure treatment improves cardiac dyssynchrony parameters in chronic systolic heart failure patients with sinus rhythm.

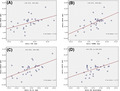

## 1 INTRODUCTION

There are cardiac conduction abnormalities in approximately 30 percent of chronic heart failure patients.^1^, ^2^ Studies show that cardiac conduction delays and dyssynchrony are important markers in the prognosis of heart failure.^1^, ^2^, ^3^ Dyssynchrony in ventricular contraction due to electrical delay leads to reduced ejection fraction and ventricular remodeling.^4^ Moreover, randomized clinical trials show that cardiac resynchronization treatment with biventricular pacing improves the clinical and structural progression of heart failure.^5^, ^6^, ^7^, ^8^

Ivabradine is a pharmacological agent which inhibits the *I*_ƒ_ channel specifically in the sinoatrial node.^9^ Its only known pharmacological effect is to slow the heart rate. In The Systolic Heart failure treatment with the *I_ƒ_* inhibitor ivabradine Trial (SHIFT), the relative risk reduction with ivabradine in the primary composite outcome of cardiovascular death or heart failure hospitalization was 18%.^10^ Also it has been shown that ivabradine treatment improves cardiac remodeling.^11^ Nevertheless, the mechanisms underlying the beneficial effects of ivabradine in heart failure are still unknown. In this study, we investigated the effect of ivabradine on cardiac dyssynchrony in patients with systolic heart failure.

## 2 METHODS

### 2.1 Patient selection

Total of thirty‐two heart failure patients referred to our study centers and who were also eligible for ivabradine treatment were enrolled in this prospective and observational study. Healthy age‐matched volunteers without heart failure were included in the control group. The criteria for inclusion were: (a) sinus rhythm, (b) LV ejection fraction ≤35%, (c) New York Heart Association (NYHA) functional class II‐IV, (d) resting heart rate ≥70 beats/min. We excluded patients with severe bradycardia due to ivabradine treatment during the study, history of cardiac resynchronization therapy (CRT) or convenient to CRT, pacemaker implantation, congenital heart disease, primary severe valve pathology, recent myocardial infarction (<2 months), and atrial fibrillation or flutter. All patients signed an informed consent form approved by our institutional review board and ethics committee. The study population was divided two groups, Group I consist of 32 reduced EF heart failure patients and Group II consist of 20 healty volunteers.

### 2.2 Medical treatment

Group I patients continued the classic heart failure treatment suggested by their doctors, including beta‐blockers, angiotensin‐converting enzyme (ACE) inhibitors, angiotensin II receptor blockers (ARB), diuretics, and digoxin. Group II patients received no medication. Ivabradine was initiated after baseline echocardiography. The starting dose was 10 mg/d (5 mg twice daily), which was uptitrated to a target dose of 15 mg/d. The use of medication was checked at 1 month and 3 months.

### 2.3 Echocardiography

All enrolled patients underwent echocardiography before and 1 month and 3 months after randomization. All imaging was performed using Vivid‐3 Pro, GE Vingmed Ultrasound in two centers and Vivid‐7, GE Vingmed Ultrasound with a 2.5 MHz probe in one center. Transthoracic echocardiography was performed by experienced echocardiography specialists who were blinded to heart failure status. An intraobserver variability study on SPWMD and Ts‐SL time among 10 volunteers showed coefficients of variation in 3.9% and 4,2%, respectively. Between observers, this coefficient was 5.6% and 6.9%. Two‐dimensional and M‐mode echocardiograms were recorded as recommended by the American Society of Echocardiography. Left ventricular end‐systolic and end‐diastolic volumes (LVESV and LVEDV) and left ventricular ejection fraction (LVEF) were calculated from apical two‐ and four‐chamber images using Simpson's biplane technique.

#### 2.3.1 Interventricular dyssynchrony

Pulsed wave Doppler was used for the measurement of interventricular dyssynchrony (IVD). The difference between preejection intervals of the left ventricular and right ventricular outflow tracts was calculated with the initiation of electrocardiographic QRS.^5^

#### 2.3.2 Intraventricular dyssynchrony

##### M‐mode echocardiography

Septal to posterior wall motion delay (SPWMD) was calculated from the parasternal long‐axis view using M‐mode echocardiography.^12^

##### Tissue Doppler imaging

Basal septal and basal lateral myocardial velocities were measured from the apical four‐chamber view. Segment time (Ts) was measured as the duration from the initiation of QRS to peak systolic myocardial velocity, and septal to lateral Ts delay (Ts‐SL) was calculated.^13^

All measurements were corrected for heart rate using Bazett's formula to avoid the confounding effect of heart rate changes. Differences between the parameters at baseline and at 3 months were calculated and recorded as “delta” (delta IVD, delta SPWMD, delta Ts‐SL).

### 2.4 Statistical analysis

The Statistical Package for Social Sciences, version 15.0 was used for statistical analysis. Data were expressed as mean ± standard deviation for continuous variables, and as numbers (percentages) for categorical variables. Kolmogorov‐Smirnov test was used to evaluate whether continuous variables were normally distributed. Group I and Group II variables were compared using Student's *t* test for continuous variables and the chi‐square test for categorical variables. Changes in echocardiographic parameters between baseline, at 1 month and 3 months in Group I were compared with repeated measurement analysis of variance. Pearson's or Spearman's correlation analysis was used to test associations between the changes in echocardiographic parameters between baseline and 3 months and the other parameters. A *P* value < .05 was considered statistically significant.

## 3 RESULTS

Fifty‐five heart failure patients were enrolled in the study, 23 of whom did not complete the study: Six were unable to come to appointments, five developed atrial fibrillation, three were unable to continue ivabradine treatment because of bradycardia (<50 beats/min), five patients died and four patients had CRT. As a result, the study included a total of 32 heart failure patients and 20 healthy volunteers. Seven patients had symptomatic bradycardia during the study and ivabradine was stopped in three patients (5.4%) due to persistent bradycardia (<50 beats/min) despite dose titration. None of the patients had a transient or permanent pacemaker.

Baseline characteristics of the patients are given in Table 1. Left ventricular volume, QRS duration and heart rate were higher in Group I than Group II, but LVEF was lower. Eighteen patients in Group I (56.3%) used ivabradine with a beta‐blocker. Of the 18 patients who received beta‐blocker treatment, 10 were prescribed metoprolol, seven were prescribed carvedilol, and one was prescribed bisoprolol. The main maintenance dose of ivabradine was 11.6 ± 3.4 mg/d. At follow‐up, there was no significant change in the other drug treatments used by the patients. The drugs used by patients in the ivabradine group at baseline, 1st month and 3rd month are given in Table 2.

**TABLE 1:** Baseline characteristics

	Group I (n = 32)	Group II (n = 20)	*P*
Age, year	63.3 ± 10.9	61.2 ± 9.3	0.479
Gender, M	25 (78.1)	15 (75.0)	0.794
Diabetes mellitus, n (%)	8 (25.0)	3 (15.0)	0.390
Smoking, n (%)	11 (34.4)	3 (15.0)	0.125
Body mass index, kg/m^2^	25.6 ± 5.0	28.8 ± 3.0	0.374
Systolic blood pressure, mmHg	122.3 ± 24.5	133.1 ± 22.6	0.054
Diastolic blood pressure, mmHg	74.3 ± 14.3	77.1 ± 12.2	0.438
QRS duration, ms	109.1 ± 21.5	88.3 ± 10.8	<0.001
Heart rate, beats/min	85.7 ± 11.9	67.4 ± 10.0	<0.001
Left bundle branch block	8 (25.0)	0	0.015
Echocardiography			
LVend‐diastolic volume, mL	201.4 ± 52.4	109.6 ± 10.4	<0.001
LVend‐systolic volume, mL	139.4 ± 42.2	43.6 ± 6.6	<0.001
LVejection fraction, %	31.3 ± 5.6	60.3 ± 6.7	<0.001
Interventricular dyssynchrony, ms	42.0 ± 24.4	22.7 ± 7.9	0.001
SPWMD, ms	90.3 ± 21.4	58.4 ± 16.6	<0.001
Ts‐SL, ms	42.7 ± 24.7	22.3 ± 7.6	0.001
Ischemic etiology, %	20 (62.5)		
Idiopathic cardiomyopathy	12 (37.5)		
Treatment			
Beta‐blocker, n (%)	18 (56.3)		
ACE inhibitor, n (%)	29 (90.6)		
ARB, n (%)	3 (9.4)		
Furosemide, n (%)	25 (78.1)		
Spironolactone, n (%)	12 (37.5)		
Digoxin, n (%)	4 (12.5)		
Ivabradine, mg daily	11.6 ± 3.4		
NYHA			
II	13 (40.6)		
III	14 (43.8)		
IV	5 (15.6)		

Note

Results are presented as mean ± standard deviation.

Abbreviations: NYHA, New York Heart Association functional class; SPWMD: Septal to posterior wall motion delay.

**TABLE 2:** Comparison of medical treatment of patients in Group I at initiation, 1 month,and 3 months

Treatment (Group I) (n = 32)	initiation	1.month	3. months	*P*
Beta‐blocker, n (%)	18 (56.3)	17 (56.3)	17 (56.3)	0.958
ACE inhibitor, n (%)	29 (90.6)	29 (90.6)	29 (90.6)	1
ARB, n (%)	3 (9.4)	3 (9.4)	3 (9.4)	1
Furosemide, n (%)	25 (78.1)	23 (78.1)	21 (78.1)	0.538
Spironolactone, n (%)	12 (37.5)	10 (37.5)	9 (37.5)	0.716
Digoxin, n (%)	4 (12.5)	3 (12.5)	1 (12.5)	0.384
Ivabradine, mg daily		11.6 ± 3.4	11.6 ± 3.4	0.969

Abbreviations: ACE, angiotensin‐converting enzyme; ARB, angiotensin receptor blocker.

### 3.1 Clinical parameters

In Group I, heart rate was 85.7 ± 11.9 beats/min at baseline, 68.1 ± 13.4 beats/min (*P* < .001) at 1 month, and 63.5 ± 11.2 beats/min at 3 months (*P* < .001). In Group I, LV ejection fraction was 31.3 ± 5.6% at baseline, 32.4 ± 5.8% (*P* < .05) at 1 month, and 34.9 ± 7.5% at 3 months (*P* < .001).In Group I, LV end‐diastolic volume was 201 ± 54mL at baseline, 193.7 ± 48.9 mL (*P* < .05) at 1 month, and 187.5 ± 48.3 mL at 3 months (*P* < .001). In Group, I LV end‐systolic volume was 139.4 ± 42.2 mL at baseline, 132.1 ± 39.2 mL (*P* < .05) at 1 month, and 123.3 ± 39.5 mL at 3 months (*P* < .001). In Group I, interventricular dyssynchrony was 42.0 ± 24.4 milliseconds at baseline, 33.6 ± 20.7 milliseconds (*P* < .05) at 1 month, and 30.7 ± 19.4 milliseconds at 3 months (*P* = .002). In Group I, septal to posterior wall motion delay was 90.3 ± 21.4 milliseconds at baseline, 83.9 ± 26.9 milliseconds (*P* < .05) at 1 month, and 81.5 ± 27.3 milliseconds at 3 months (*P* = .001). In Group I, septal to lateral Ts delay was 42.7 ± 24.5 milliseconds at baseline, 35.8 ± 22.6 milliseconds (*P* < .05) at 1 month, and 34.8 ± 22.4 milliseconds at 3 months (*P* = .002). In Group I, there were 19 NYHA III–IV class patients at baseline, 8 at 1 month (*P* = .011), and 3 at 3 months (*P* = .002) (Table 3).

**TABLE 3:** Comparison of the effects of ivabradine on left ventricular reverse remodeling, dyssynchrony parameters and hemodynamics at 1 month and 3 months

	Group I			*P*
	Baseline	1 month	3 months	
LVejection fraction, %	31.3 ± 5.6	32.4 ± 5.8*	34.9 ± 7.5*	0.001
LVend‐diastolic volume, mL	201.4 ± 52.4	193.7 ± 48.9*	187.5 ± 48.3^*,†^	<0.001
LVend‐systolic volume, mL	139.4 ± 42.2	132.1 ± 39.2*	123.3 ± 39.5^*,†^	<0.001
Heart rate (beat/min)	85.7 ± 11.9	68.1 ± 13.4*	63.5 ± 11.2*	<0.001
Interventricular dyssynchrony, ms	42.0 ± 24.4	33.6 ± 20.7*	30.7 ± 19.4^*,†^	0.002
SPWMD (ms)	90.3 ± 21.4	83.9 ± 26.9*	81.5 ± 27.3^*,†^	0.001
Ts‐SL, ms	42.7 ± 24.5	35.8 ± 22.6*	34.8 ± 22.4*	0.002
NYHA 3‐4, %	19 (59)	8 (25)*	6 (19)*	0.001

* *P* < .05 comparison with baseline.

^†^ *P* < .05 comparison with 1 month.

### 3.2 Intraventricular dyssynchrony

Baseline SPWMD and Ts‐SL values were significantly higher in Group I than in Group II (respectively, *P* < .001 and *P* = .001) (Table 1). There was a clear reduction in SPWMD and Ts‐SL after 1 month and 3 months of ivabradine treatment. SPMWD was 90.3 ± 21.4 milliseconds at baseline, 83.9 ± 26.9 milliseconds at 1 month (*P* = .011), and 81.5 ± 27.3 milliseconds (*P* = .001) at 3 months. Ts‐SL was 42.7 ± 24.5 milliseconds at baseline, 35.8 ± 22.6 milliseconds at 1 month (*P* < .001), and 34.8 ± 22.4 milliseconds at 3 months (*P* = .002) (Table 3).

### 3.3 Interventricular dyssynchrony

IVD was greater in Group I than in Group II (42.0 ± 24.4 milliseconds vs 22.7 ± 7.9 milliseconds, *P* = .001) at baseline, and had decreased to 33.6 ± 20.7 milliseconds (*P* = .001) at 1 month and 30.7 ± 19.4 milliseconds (*P* < .001) at 3 months (Table 3).

### 3.4 LV Reverse remodeling

**FIGURE 1:**
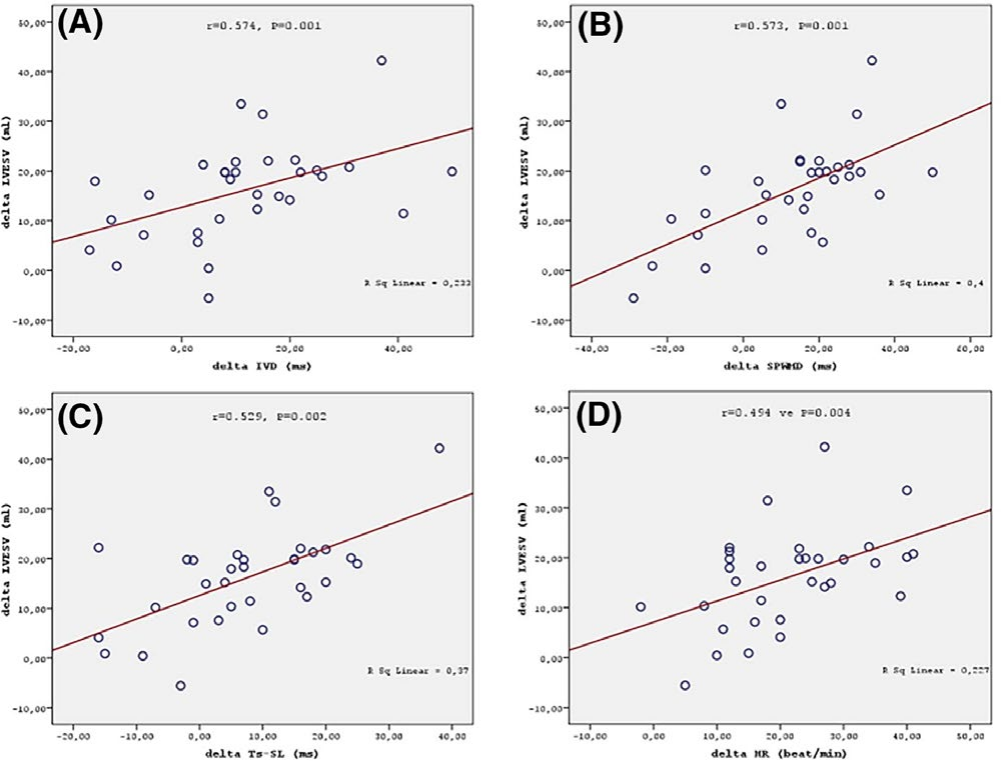
Correlation between delta LVESV and delta IVD (A), Correlation between delta LVESV and delta SPWMD (B), Correlation between delta LVESV and delta Ts‐SL (C), Correlation between delta LVESV and delta HR (D)

Baseline LVEF, LVEDV, and LVESV values in Group I differed significantly from those in Group II (*P* < .001). LVEF increased from 31.3 ± 5.6% at baseline to 32.0 ± 5.6% at 1 month (*P* = .061) and 34.9 ± 7.5% (*P* < .001) at 3 months. LVEDV decreased from 201.4 ± 52.4 mL to 195.9 ± 50.7 mL at 1 month (*P* = .103) and 187.5 ± 48.3 mL (*P* < .001) at 3 months. LVESV decreased from 139.4 ± 42.2 mL to 135.3 ± 39.6 mL (*P* = .006) at 1 month and 123.3 ± 39.5 mL (*P* < .001) at 3 months (Table 3). There was significant correlation between the change in LVESV (delta LVESV) and dyssynchrony parameters. The correlations of delta LVESV with delta IVD (r = 0.574, *P* = .001), delta SPWMD (r = 0.573, *P* = .001), delta Ts‐SL (r = 0.529, *P* = .002), and delta HR (r = 0.494, *P* = .004) were significant (Figure 1).

## 4 DISCUSSION

This study demonstrates that the addition of ivabradine to heart failure treatment improves cardiac dyssynchrony in chronic systolic heart failure patients with sinus rhythm. This improvement is related to left ventricular ejection fraction, functional capacity, and left ventricular reverse remodeling. This is the first study showing a relationship between ivabradine and cardiac dyssynchrony.

Most heart failure patients have conduction abnormalities ^1^, ^2^. Dyssynchrony in ventricular contraction due to electrical delay reduces ejection fraction and induces ventricular remodeling ^3^. Recent studies show that cardiac dyssynchrony and prolonged QRS duration worsen the prognosis of heart failure ^14^, ^15^. On the other hand, cardiac resynchronization treatment with biventricular pacing improved symptoms, quality of life, exercise capacity, left ventricular systolic function, and left ventricular remodeling in randomized clinical trials.^5^, ^6^, ^7^, ^8^ In our study, intraventricular and interventricular dyssynchrony were higher in heart failure patients than in the control group. Also, QRS duration was longer in heart failure patients than in the control group.

Ivabradine is a pharmacological agent which inhibits the *I*_ƒ_ channel specifically in the sinoatrial node.^9^ Its only known pharmacological effect is to slow the heart rate. The SHIFT study enrolled 6588 heart failure patients in sinus rhythm with NYHA functional class II‐IV and EF ≤35%. Treatment with ivabradine superimposed on background therapy for heart failure was associated with an 18% reduction in risk for the primary composite endpoint of cardiovascular death or hospitalization for worsening heart failure (*P < *.00001).^10^ Volterrani et al^16^ reported that ivabradine treatment alone or in combination with carvedilol is more effective than carvedilol alone at improving exercise tolerance and quality of life in heart failure patients. Also, the recent European Society of Cardiology heart failure treatment guidelines suggest adding ivabradine to the medication of patients in sinus rhythm and a heart rate >70 beats/min with symptoms that persist despite standard medical treatment.^17^

Cardiac remodeling is a central feature of the progression of heart failure. In our study, the addition of ivabradine to heart failure treatment led to improvement in cardiac remodeling. Our results are evidence of the relationship between heart rate and cardiac remodeling. Also in a subgroup analysis of the SHIFT study, ivabradine treatment improved cardiac remodeling.^11^ During 8 months of follow‐up in the ivabradine group, reduction in LV end‐systolic and end‐diastolic volumes and increase in LVEF were greater than in the placebo group. In a rat model of chronic mild heart failure, ivabradine preserved cardiac output and improved left ventricular function and geometry. These changes were linked to modifications in the extracellular matrix and in cardiac myocyte function.^18^ Becher et al suggested that the protection against cardiac fibrosis and remodeling with ivabradine was greater than that afforded by metoprolol in an animal model of experimental heart failure.^19^ Similar effects with ivabradine have been found by other researchers in a rat model of chronic severe heart failure, including reductions in fibrosis, local RAAS stimulation, and sympathetic drive.^20^, ^21^, ^22^

In our study, the reduction in heart rate was related to improvement in cardiac dyssynchrony parameters in chronic heart failure patients with sinus rhythm. Beta‐blockers have been shown to improve cardiac dyssynchrony in a limited number of studies^23^, ^24^, ^25^ in which the relationship between left ventricular remodeling and heart rate was not elucidated. However, Ishii et al^26^ reported that heart rate was the only independent predictor of left ventricular reverse remodeling in heart failure patients. In a rat model of left ventricular dysfunction created with myocardial infarction, cardiac remodeling and action potential duration were preserved in the ivabradine group when compared with the control group.^27^ In a study evaluating the effect of ivabradine on dyssynchrony parameters, an improvement in “Ts” was found correlated with our study, but interventricular dyssynchrony and SPWMD were not evaluated in this study.^28^ In our study, the increase in LVEF was related to heart rate reduction and improvement in cardiac dyssynchrony. Also, the changes in LVEF and dyssynchrony suggest that electrical remodeling improves faster than structural remodeling. Structural remodeling is therefore thought to be improved after electrical remodeling.

### 4.1 Study limitation

Ours was an observational study in a limited number of heart failure patients. Also the etiologies of heart failure and QRS duration were not homogeneous in the patients. Moreover, the lack of a heart failure group which was not treated with ivabradine renders the attribution of improvement in dyssynchrony to ivabradine more difficult.

## 5 CONCLUSION

This study shows that effective heart rate reduction with ivabradine treatment improves cardiac dyssynchrony and cardiac remodeling. Cardiac electrical remodeling accompanied by heart rate reduction could explain the effect of ivabradine in the treatment of heart failure. However, these findings need to be validated in larger and more homogeneous patient groups.

## CONFLICT OF INTEREST

None declared.
